# Tnfa Signaling Through Tnfr2 Protects Skin Against Oxidative Stress–Induced Inflammation

**DOI:** 10.1371/journal.pbio.1001855

**Published:** 2014-05-06

**Authors:** Sergio Candel, Sofía de Oliveira, Azucena López-Muñoz, Diana García-Moreno, Raquel Espín-Palazón, Sylwia D. Tyrkalska, María L. Cayuela, Stephen A. Renshaw, Raúl Corbalán-Vélez, Inmaculada Vidal-Abarca, Huai-Jen Tsai, José Meseguer, María P. Sepulcre, Victoriano Mulero

**Affiliations:** 1 Departamento de Biología Celular e Histología, Facultad de Biología, Universidad de Murcia, Murcia, Spain; 2 Instituto Murciano de Investigación Biosanitaria (IMIB), Murcia, Spain; 3 Carlota Saldanha Lab, Instituto de Medicina Molecular, Instituto de Bioquímica, Faculdade de Medicina, Universidade de Lisboa, Lisboa, Portugal; 4 Instituto de Investigaciones Marinas, Consejo Superior de Investigaciones Científicas (CSIC), Vigo, Spain; 5 Grupo de Telómeros, Envejecimiento y Cáncer, Unidad de Investigación, Departamento de Cirugía, CIBERehd. Hospital Universitario “Virgen de la Arrixaca,” Murcia, Spain; 6 MRC Centre for Developmental and Biomedical Genetics, University of Sheffield, Sheffield, United Kingdom; 7 Servicio de Dermatología, Hospital Universitario “Virgen de la Arrixaca,” Murcia, Spain; 8 Servicio de Anatomía Patológica, Hospital Universitario “Virgen de la Arrixaca,” Murcia, Spain; 9 Institute of Molecular and Cellular Biology, National Taiwan University, Taipei, Taiwan; St. Jude Children's Research Hospital, United States of America

## Abstract

TNFα overexpression has been associated with several chronic inflammatory diseases, including psoriasis, lichen planus, rheumatoid arthritis, and inflammatory bowel disease. Paradoxically, numerous studies have reported new-onset psoriasis and lichen planus following TNFα antagonist therapy. Here, we show that genetic inhibition of Tnfa and Tnfr2 in zebrafish results in the mobilization of neutrophils to the skin. Using combinations of fluorescent reporter transgenes, fluorescence microscopy, and flow cytometry, we identified the local production of dual oxidase 1 (Duox1)-derived H_2_O_2_ by Tnfa- and Tnfr2-deficient keratinocytes as a trigger for the activation of the master inflammation transcription factor NF-κB, which then promotes the induction of genes encoding pro-inflammatory molecules. In addition, pharmacological inhibition of Duox1 completely abrogated skin inflammation, placing Duox1-derived H_2_O_2_ upstream of this positive feedback inflammatory loop. Strikingly, DUOX1 was drastically induced in the skin lesions of psoriasis and lichen planus patients. These results reveal a crucial role for TNFα/TNFR2 axis in the protection of the skin against DUOX1-mediated oxidative stress and could establish new therapeutic targets for skin inflammatory disorders.

## Abstract 2

A new zebrafish model of skin inflammatory disease explains new-onset and worsening psoriasis and lichen planus in patients receiving anti-TNFα therapy.

## Author Summary

Psoriasis and lichen planus are chronic, debilitating skin diseases that affect millions of people worldwide. TNFα is a multifunctional cytokine that mediates acute and chronic inflammation. While TNFα antagonist therapy is used for autoimmune or chronic inflammatory diseases, such as inflammatory bowel disease (IBD), numerous studies have reported new-onset psoriasis and lichen planus following such therapy. We have used the unique advantages of the zebrafish embryo to identify a novel phenotype that mirrors this unexplained and paradoxical onset of psoriasis and lichen planus. We found that depletion of Tnfa or its receptor Tnfr2 caused skin inflammation and hyperproliferation of keratinocytes through the activation of a Duox1/H_2_O_2_/NF-κB positive feedback inflammatory loop. Strikingly, DUOX1 was drastically induced in the skin lesions of psoriasis and lichen planus patients, and pharmacological inhibition of Duox1 abrogated skin inflammation, placing Duox1-derived H_2_O_2_ upstream of this inflammatory loop. Our results suggest that therapies targeting DUOX1 and H_2_O_2_ could provide innovative approaches to the management of skin inflammatory disorders.

## Introduction

Tumor necrosis factor α (TNFα) is a multifunctional cytokine that mediates key roles in acute and chronic inflammation, antitumor responses, and infection. TNFα binds TNF receptor 1 (TNFR1, also known as TNFRSF1A or P55) and TNFR2 (also known as TNFRSF1B or P75) for stimulation of two opposing signaling events [1]. In general, TNFR1 signaling results in the trigger of a cascade that can result in apoptosis [2]. This is dependent upon the cell type, the state of activation of the cell, and the cell cycle. In contrast, a TNFR2 signal induces cell survival pathways that can result in cell proliferation [2].

Enhanced TNFα synthesis is associated with the development of autoimmune/chronic inflammatory diseases, including psoriasis, lichen planus, rheumatoid arthritis, and inflammatory bowel disease (IBD). The inhibition of TNFα activities in these diseases has been remarkably successful [3],[4]. Paradoxically, however, numerous studies have reported new-onset psoriasis and lichen planus, or worsening of existing psoriasis, following TNFα antagonist therapy in adult patients [5]–[10]. Despite these clinical data pointing to an ambiguous function of TNFα in psoriasis and lichen planus, the role of TNFα, and in particular the contribution of each TNFR, in the regulation of skin inflammation has been scarcely studied. An earlier study using gene-targeted mutant mice lacking either TNFR1 or TNFR2 showed that skin inflammation induced indirectly by irritant chemicals or directly by intradermal administration of TNFα was greatly attenuated in TNFR1-deficient mice, whereas TNFR2-deficient siblings responded normally [11]. In addition, mice with an arrested canonical NF-κB activation pathway in the keratinocytes develop a severe inflammatory skin disease shortly after birth, which is caused by TNFα- and macrophage-mediated, but T-cell–independent, mechanisms [12]–[16]. The characteristics of this complex disorder are strikingly similar to those associated with the human X-linked genodermatosis incontinentia pigmenti (IP) [17]. To the best of our knowledge, however, the role played by TNFα in the homeostasis of healthy skin has never been studied.

TNFα and TNFRs are conserved in all vertebrates. Recent studies have shown that in the zebrafish (*Danio rerio*) Tnfa functions as a pro-inflammatory cytokine [18] and Tnfr signaling plays an important role in the homeostasis of endothelial cells [19]. In the present study, we have taken advantage of the strengths of the zebrafish embryo model to study the impact of Tnfa, Tnfr1, and Tnfr2 deficiencies in a whole vertebrate organism. We found that Tnfa and Tnfr2 are both crucial, whereas Tnfr1 is dispensable, for the homeostasis of the skin. Genetic inhibition of Tnfa and Tnfr2 promotes H_2_O_2_-mediated skin infiltration by neutrophils, increased keratinocyte proliferation, and the local activation of the master inflammation transcription factor NF-κB, which then promotes the induction of genes encoding pro-inflammatory molecules. In addition, DUOX1 was strongly induced in keratinocytes of human psoriasis and lichen planus patients.

## Results

### Tnfa or Tnfr2 Deficiency Results in Neutrophil Mobilization to the Skin

**Figure 1:**
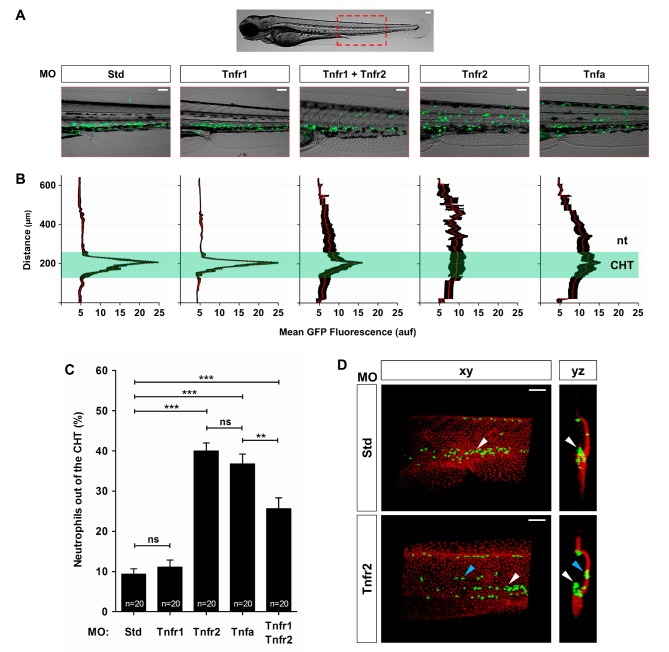
TNFα and Tnfr2 deficiencies result in neutrophil mobilization to the skin. Zebrafish one-cell *mpx:eGFP* and/or *krt18:RFP* embryos were injected with standard control (Std), Tnfr1, Tnfr2, Tnfa, or Tnfr1+Tnfr2 MOs. (A) Representative images, bright field and green channels, of the morphants at 3 dpf showing the differences in the neutrophils distribution. (B) Fluorescence intensity was measured for all the groups in the area indicated (A), which includes the CHT, where most neutrophils are located in wild-type larvae at 3 dpf. The images were converted to a fluorescence value matrix where the value obtained for each pixel transversally was the mean ± S.E.M. for all the pixels for each row (15 larvae per treatment from 3 different experiments). The area corresponding to the CHT has been labeled and highlighted. The notochord (nt) location has been indicated to facilitate the larval orientation. auf, arbitrary units of fluorescence. (C) The neutrophil mobilization from the CHT in Tnfa- and Tnfr2-deficient larvae was quantified as the percentage of neutrophils outside the CHT in 20 larvae per group from 3 different experiments. The mean ± S.E.M. for each group is shown. (D) Representative frontal (*xy*) and lateral (*yz*) views of tridimensional reconstructions from confocal microscopy images of WIHC of *mpx:eGFP* larvae stained at 3 dpf with anti-p63 antibodies (basal keratinocyte marker, red) showing the neutrophils' distribution in the CHT area of control and Tnfr2-deficient larvae. Note that most neutrophils (eGFP, green) are located in the CHT in control larvae (white arrowheads), while many of them infiltrate the skin (blue arrowheads) of Tnfr2-deficient larvae, whereas they are mainly located in the CHT in their wild-type siblings. Scale bars, 100 µm. ns, not significant. *p<0.05, **p<0.01, ***p<0.001.

In wild-type larvae, most neutrophils (approximately 90%) were located in the caudal hematopoietic tissue (CHT) [20] by 3 d postfertilization (dpf) (Figure 1A–C), which is consistent with neutrophil localization patterns described previously [21],[22]. However, in Tnfa- and Tnfr2-deficient larvae, approximately 40% of neutrophils were located outside the CHT (Figure 1A–C). In addition, Tnfr1-deficient animals showed a normal neutrophil distribution, whereas their double deficient siblings for both Tnfr1 and Tnfr2 showed also a distribution pattern more similar to single Tnfr2-deficient fish (Figure 1A–C). The specificity of this phenotype was confirmed with a dominant negative (DN) Tnfr2, which is lacking the entire intracellular signaling domain, but is identical to full-length Tnfr2 in its transmembrane and extracellular domains, and therefore, its trimerization with endogenous Tnfr2 extinguishes Tnfr2 signaling [19]. The results showed that the altered neutrophil distribution of Tnfr2 morphants was phenocopied by overexpression of DN-Tnfr2 (Figure S1A). In addition, the scattered distribution of Tnfa- and Tnfr2-deficient larvae was partially rescued by overexpression of wild-type Tnfa and Tnfr2 RNAs, respectively (Figure S1B). These results prompted us to examine the distribution of macrophages in TNFα- and TNFR2-deficient fish, and surprisingly, macrophage distribution was apparently normal in all cases (Figure S2).

To ascertain the precise localization of neutrophils in Tnfa/Tnfr2-deficient larvae, we knocked down Tnfr2 in transgenic *mpx:eGFP* animals followed by whole mount immunohistochemistry (WIHC) against p63 (basal keratinocyte marker) to visualize neutrophils (GFP^+^) and skin keratinocytes (p63^+^) at the same time in whole larvae. The results revealed that although neutrophils from wild-type animals were mainly located in the CHT, a high proportion of neutrophils were seen in close contact with keratinocytes in Tnfr2-deficient animals (Figure 1D and Videos S1 and S2). Collectively, these results indicate that deficiency of either Tnfa or Tnfr2 specifically promotes neutrophil infiltration into the skin of zebrafish during early development.

### Tnfa or Tnfr2 Deficiency Triggers the Induction of Genes Encoding Pro-Inflammatory Mediators in Keratinocytes

**Figure 2:**
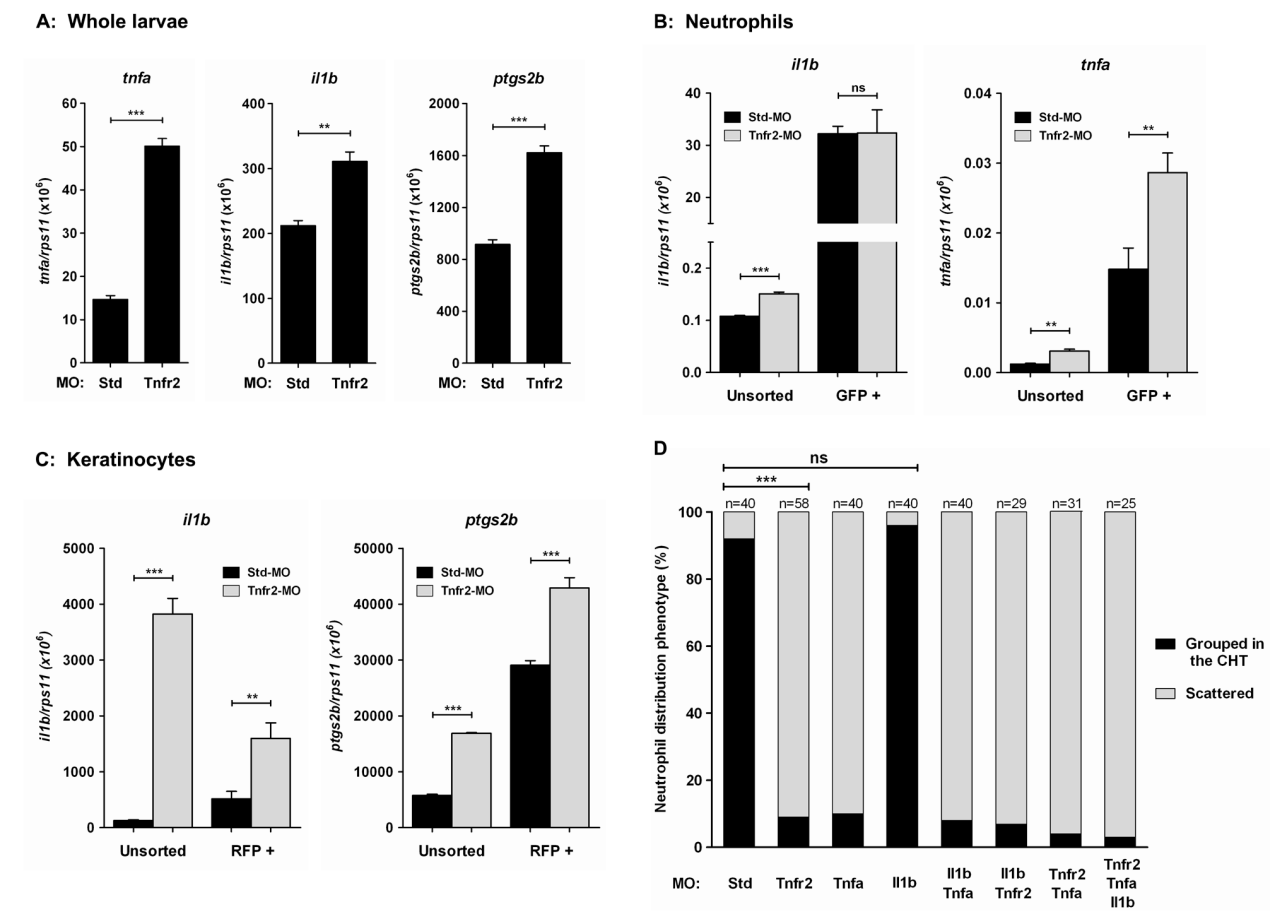
TNFa and Tnfr2 deficiencies trigger skin inflammation. Zebrafish one-cell *mpx:eGFP* or *krt18:RFP* embryos were injected with standard control (Std), Tnfr2, Il1b, and/or Tnfa MOs. The expression of *tnfa*, *il1b*, and *ptgs2b* genes was measured by RT-qPCR in whole body (A), FACS-sorted neutrophils (B), and FACS-sorted keratinocytes (C) from Std and Tnfr2 morphants at 3 dpf. (D) The phenotype of 3 dpf morphant larvae was classified as neutrophils grouped in the CHT or scattered, as described in Figure 1. Note that IL-1β knockdown failed to rescue the neutrophil mobilization in Tnfr2-deficient larvae. ns, not significant. *p<0.05; **p<0.01; ***p<0.001.

The phenotype of Tnfa- and Tnfr2-deficient fish is reminiscent of that of *spint1a* and *clint1* mutant fish, which show chronic skin inflammation characterized by increased interleukin-1β (IL-1β) production and neutrophil infiltration [23]–[25]. This led us to examine the expression of three genes encoding major pro-inflammatory molecules, namely Tnfa itself, IL-1β, and prostaglandin-endoperoxide synthase 2b (PTGS2b, also known as COX2b), in whole wild-type and Tnfr2-deficient larvae at 3 dpf as well as in sorted *mpx:eGFP* ^+^ cells—that is, neutrophils. It was found that Tnfr2 deficiency triggered the expression of *tnfa*, *il1b*, and *ptgs2b* genes (Figure 2A). Although neutrophils highly expressed the genes encoding Tnfa and Il1b as well as both Tnfrs, they did not mediate the induction of *il1b* observed in Tnfr2-deficient fish (Figures 2B, S3A, and S4A). Nevertheless, the transcript levels of *tnfa* were higher in neutrophils from Tnfr2-deficient fish than in neutrophils from their wild-type siblings (Figure 2B), but this might reflect a positive feedback loop in response to Tnfr2 deficiency [19]. In addition, Tnfr2-deficient embryos showed higher transcript levels of *il1b* at 24 hpf (Figure S5), soon after the development of the first neutrophils in the zebrafish embryo [21],[22],[26] and before hatching. We then sorted krt18^+^ cells from Tnfa- and Tnfr2-deficient animals at 3 dpf and found that they show much higher transcript levels of *il1b* and *ptgs2b* than krt18^+^ cells from wild-type animals (Figures 2C and S3B). Notably, krt18^+^ cells expressed both Tnf receptors (Figure S4B) and the specific marker of basal keratinocytes p63 (Figure S3B).

We next wondered whether knockdown of Il1b using a specific morpholino (MO) [27] might rescue the neutrophil dispersion of Tnfr2-deficient animals. As shown in Figure 2D, genetic inhibition of Il1b failed to rescue the neutrophil dispersion observed in Tnfr2 morphants. These results taken together indicate that the Tnfa/Tnfr2 axis is required for skin homeostasis in zebrafish and that the deficiency of either ligand or receptor triggers an inflammatory response characterized by the induction of pro-inflammatory mediators and neutrophil infiltration.

### Tnfa and Tnfr2 Deficiencies Induce NF-κB Activation in the Skin

**Figure 3:**
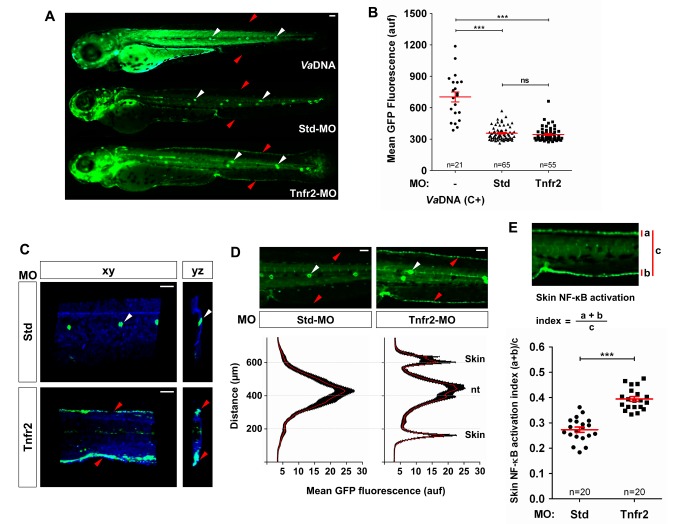
Tnfa and Tnfr2 deficiencies result in skin NF-κB activation. Zebrafish one-cell *NF-κB:eGFP* (A, B, D, E) or *NF-κB:eGFP*; *krt18:RFP* (C) embryos were injected with standard control (Std) or Tnfr2 MOs alone or in the presence of 2.3 ng/egg of *V. anguillarum* genomic DNA (VaDNA), as a positive control for NF-κB activation. (A) Representative pictures showing the induction of NF-κB activation in the skin (red arrowheads) of Tnfr2-deficient larvae at 72 hpf and the ubiquitous, strong induction in their VaDNA-injected siblings. Note the strong expression of NF-κB in neuromasts of control larvae (white arrowheads). (B) The mean GFP fluorescence was quantified in whole larvae, and no significant differences between Tnfr2-morphants and control larvae were observed. Each dot represents the mean GFP fluorescence per single larva. The mean ± S.E.M. of the whole GFP fluorescence for each group of larvae is also shown. (C) Representative frontal (*xy*) and lateral (*yz*) views of tridimensional reconstructions from confocal microscopy images of WIHC of *NF-κB:eGFP*; *krt18:RFP* larvae stained at 3 dpf with anti-RFP antibodies (keratinocytes, blue) showing the induction of NF-κB in the skin (eGFP, green) of Tnfr2-deficient larvae. (D, E) Quantification of NF-κB activation in the skin of Tnfr2-deficient larvae at 72 hpf. (D) Fluorescence intensity was measured in the area indicated of wild-type and Tnfr2-deficient larvae, as explained in the legend to Figure 1 (15 larvae per treatment from 3 different experiments). The skin and the neuromasts have been labeled to facilitate the larval orientation. Note the activation of NF-κB in the skin of Tnfr2-deficient larvae. (E) The skin NF-κB activation index was defined as the fluorescence in the skin (a+b) relative to the total fluorescence of the whole larvae (c). Each dot represents the skin NF-κB activation index per single larva. The mean ± S.E.M. of the skin NF-κB activation index for each group of larvae is also shown. Scale bars, 100 µm. ns, not significant; auf, arbitrary units of fluorescence. *p<0.05; **p<0.01; ***p<0.001.

**Figure 4:**
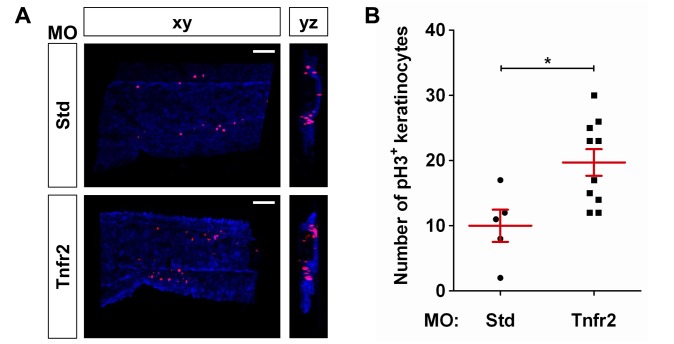
Tnfr2 deficiency results in increased proliferation of skin keratinocytes. Zebrafish one-cell *krt18:RFP* embryos were injected with standard control (Std) or Tnfr2 MOs. (A) Representative frontal (*xy*) and lateral (*yz*) tridimensional reconstructions from confocal microscopy images of WIHC of *krt18:RFP* larvae stained at 3 dpf with anti-RFP (keratinocytes, blue) and anti-phosphorylated H3 (pH 3, proliferation marker) antibodies. (B) Quantification of the number of pH3/RFP^+^ (i.e., proliferating keratinocytes) cells in the CHT area. Each dot represents one single larva, and the mean ± S.E.M. for each group of larvae is also shown. Scale bars, 100 µm. *p<0.05.

The master regulator of inflammation NF-κB plays an essential role in the homeostasis of skin. Thus, genetic inhibition of the NF-κB pathway in keratinocytes triggers a severe inflammatory skin disease in newborn mice, which is completely rescued by Tnfa and Tnfr1 depletion [12]–[16]. We therefore use a NF-κB reporter line [28] to visualize the dynamics of NF-κB in whole Tnfr2-deficient larvae. Injection of bacterial DNA, which activated TLR9, resulted in a drastic activation of NF-κB in the whole larvae (Figure 3A–B), as expected from previous results [29],[30]. Interestingly, Tnfr2 deficiency promoted a restricted activation of NF-κB in the skin (Figure 3A–E and Videos S3 and S4). Furthermore, although skin integrity was unaffected up to 5 dpf in Tnfr2-deficient larvae, as assayed by histology (Figure S6), they showed increased keratinocyte proliferation, as assayed in double transgenic NF-κB:eGFP; *krt18:RFP* animals and double WIHC with anti-RFP and anti-phosphorylated H3 (Figure 4).

### Tnfa and Tnfr2 Deficiencies Trigger H_2_O_2_ Production in the Skin

**Figure 5:**
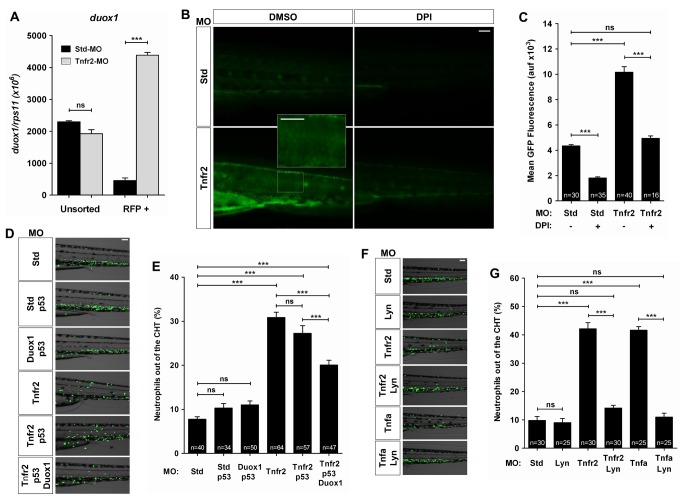
Tnfa and Tnfr2 deficiencies result in the Duox1-derived H_2_O_2_ production by keratinocytes. Zebrafish one-cell *krt18:RFP* (A), wild-type (B, C), or *mpx:eGFP* (D–G) embryos were injected with standard control (Std), Tnfr2, Tnfa, Duox1/p53, and/or Lyn MOs. (A) The expression of the *duox1* gene was measured by RT-qPCR in FACS-sorted keratinocytes from 72 hpf wild-type and Tnfr2-deficient larvae. (B, C) Wild-type and Tnfr2-deficient larvae were dechorionated at 24 hpf and treated by immersion in 100 µM DPI or vehicle alone (DMSO) for 24 h and then labeled with 50 µM acetyl-pentafluorobenzene sulphonyl fluorescein. Representative images of green channels of Std and Tnfr2 morphants are shown. Note that single keratinocytes are labeled with the H_2_O_2_ probe in Tnfr2-deficient larvae (inset). (D–G) Rescues with Duox1 (D, E) and Lyn (F, G) MOs at 72 hpf. The differences in the neutrophil distribution (D, F) and quantification of neutrophil mobilization from the CHT to the skin in the indicated number of larvae per group from three different experiments (E, G) are shown. The mean ± S.E.M. for each group is shown. Scale bars, 100 µm. ns, not significant. ***p<0.001.

Hydrogen peroxide gradients were recently shown to contribute to the early influx of neutrophils in wound [31] and tumor [32]. Interestingly, however, H_2_O_2_ is not required for neutrophil detection of localized infection [33]. These gradients are created by the dual oxidase 1 (Duox1) [31] and sensed by neutrophils through the tyrosine kinase Lyn [34]. Although identified and best studied in the zebrafish, H_2_O_2_ is likely to play the same function in human neutrophils [34]. We first analyzed the expression of the gene encoding Duox1 and found that Tnfr2-deficient keratinocytes showed higher transcript levels of *duox1* than wild-type animals (Figure 5A). Next, using an H_2_O_2_-detecting fluorescence probe, we observed that Tnfr2-deficient larvae also produced H_2_O_2_ in the skin (Figure 5B,C). We observed similar levels of labeling with the H_2_O_2_ probe in Tnfr2-deficient keratinocytes and in local keratinocytes after wounding (Figure S7). Notably, H_2_O_2_ production by Tnfr2-deficient keratinocytes preceded the activation of NF-κB (Figure S8). Consistent with these observations, genetic inhibition of Duox1 with a specific MO [31] was able to partially prevent the infiltration of neutrophils into the skin of Tnfr2-deficient larvae (Figure 5D,E). To further confirm this result, we designed a DN form of Duox1 [35], and notably, overexpression of DN-Duox1 was also able to partially prevent neutrophil infiltration in Tnfr2-deficient larvae (Figure S9A,B). Furthermore, we knocked down the H_2_O_2_ sensor of neutrophils, Lyn [34], and found full prevention of neutrophil infiltration in both Tnfr2- and Tnfa-deficient animals (Figure 5F,G).

### Pharmacological Inhibition of Duox1 Restores Skin Homeostasis in Tnfa- and Tnfr2-Deficient Animals

**Figure 6:**
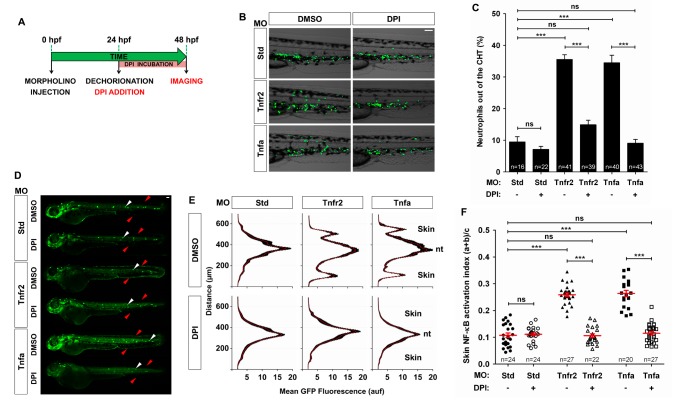
Pharmacological inhibition of Duox1 prevents skin inflammation in Tnfa- and Tnfr2-deficient zebrafish. Zebrafish one-cell *mpx:eGFP* (B, C) and *NF-κB:eGFP* (D–F) embryos were injected with standard control (Std), Tnfr2, or Tnfa MOs. (A) Scheme showing the experimental procedure: embryos were dechorionated at 24 hpf and treated by immersion in 100 µM DPI or vehicle alone (DMSO) for 24 h. (B, C) Representative images of bright field and green channels of the morphants at 48 hpf showing the differences in the neutrophils distribution (B) and quantification of neutrophil mobilization from the CHT to the skin in the indicated number of larvae per group from three different experiments (C). (D–F) Quantification of NF-κB activation in the skin of Tnfr2- and Tnfa-deficient larvae at 72 hpf. (E) Fluorescence intensity was measured for all the groups in the area indicated, as explained in the legend to Figure 1 (15 larvae per treatment from 3 different experiments). The skin and the notochord (nt) have been labeled to facilitate the larval orientation. Note the activation of NF-κB in the skin (red arrowheads) of Tnfr2-deficient larvae. Note the strong expression of NF-κB in neuromasts (white arrowheads). (F) Skin NF-κB activation index was defined as the fluorescence in the skin (a+b) relative to the total fluorescence of the whole larvae (c). Each dot represents the skin NF-κB activation index per single larva. The mean ± S.E.M. of the skin NF-κB activation index for each group of larvae is also shown. Scale bars, 100 µm. ns, not significant. ***p<0.001.

The above results prompted us to evaluate whether pharmacological inhibition of Duox1 using the NADPH oxidase inhibitor dibenziodolium chloride (DPI), which has been shown to inhibit Duox1 and H_2_O_2_ gradient formation in zebrafish [31],[34], may restore skin homeostasis in Tnfa- and Tnfr2-deficient larvae. The results showed that DPI treatment completely inhibited the generation of H_2_O_2_ in the skin (Figure 5B,C), the infiltration of neutrophils (Figure 6A–C) into this tissue, and more importantly, skin NF-κB activation (Figure 6D–F) in both Tnfa- and Tnfr2-deficient animals. Collectively, these results demonstrate that the Tnfa/Tnfr2 axis is indispensable for skin homeostasis and its inhibition results in the release of Duox1-derived H_2_O_2_, local activation of NF-κB, induction of genes encoding Duox1 and pro-inflammatory mediators, and neutrophil infiltration.

### DUOX1 Is Induced in Human Psoriasis and Lichen Planus Lesions

**Figure 7:**
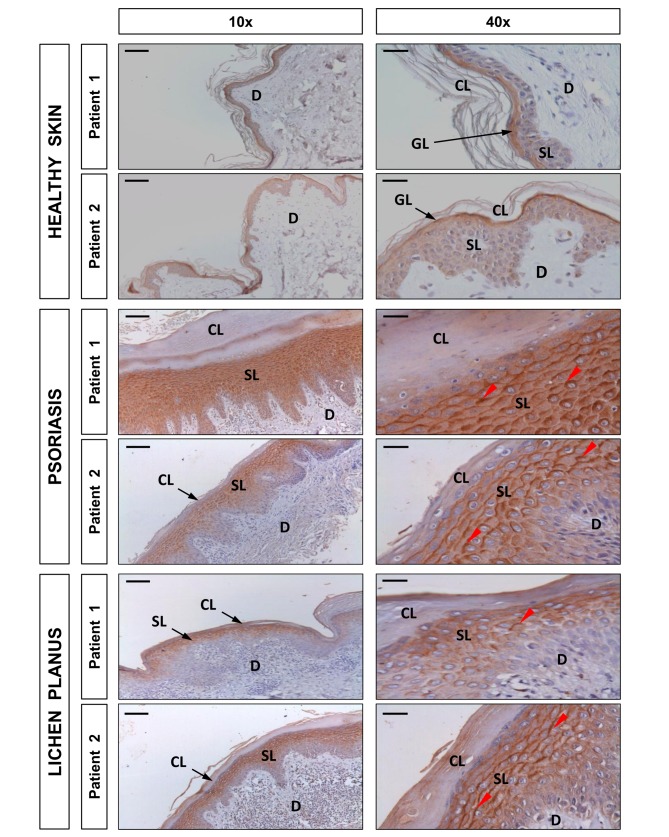
DUOX1 is induced in human psoriasis and lichen planus lesions. Representative images of sections from two healthy, two psoriatic, and two lichen planus skin biopsies that have been immunostained with an anti-DUOX1 goat polyclonal antibody and then slightly counterstained with hematoxilin. Note that DUOX1 is weakly expressed in healthy epidermis, mainly in the granular layer (GL), whereas it is strongly expressed (red arrowheads) in the spinous layer (SL) of both psoriasis and lichen planus lesions. CL, cornified layer; D, dermis. Scale bars, 100 µm (left panel) and 30 µm (right panel).

The crucial role of Duox1-generated H_2_O_2_ in the infiltration of neutrophils into the skin and the induction of NF-κB prompted us to investigate if this inflammatory signal may also play a role in human psoriasis and lichen planus. We analyzed by immunohistochemistry 10 healthy skins and 8 lichen planus and 15 psoriasis lesions using an antibody to human DUOX1 (Figure 7). The results showed that although DUOX1 was expressed at low levels in healthy epidermis, mainly in the granular layer, a drastic induction of this enzyme was obvious in the keratinocytes of the spinous layer of the epidermis from both psoriasis and lichen planus lesions. In some patients, the induction was obvious in all keratinocytes of the spinous layer, whereas in others it was observed only in the upper layers of this stratum. It was noticeable the localization of DUOX1 in the plasma membrane of psoriasis and lichen planus keratinocytes and also in their cytoplasm, where it was accumulated in the upper side of these cells—that is, facing the cornified layer. Although this particular distribution deserves further investigation, these results strongly suggest a role for DUOX1 in psoriasis and lichen planus.

## Discussion

**Figure 8:**
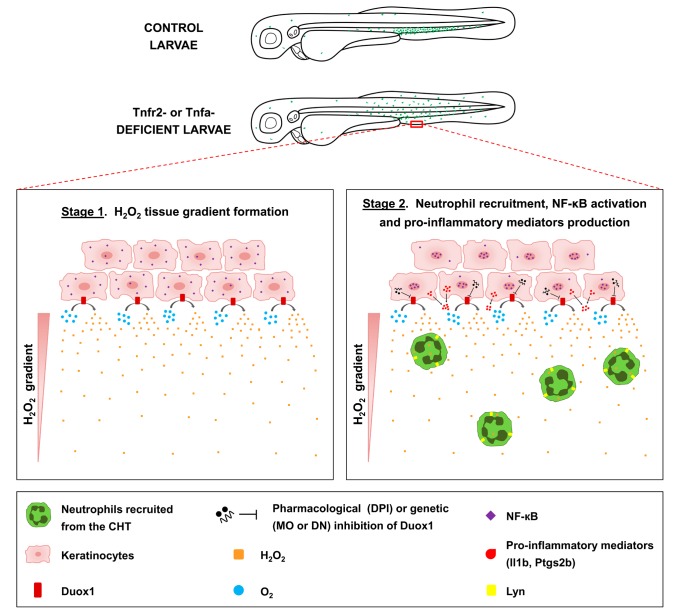
Proposed model illustrating the H_2_O_2_/NF-κB/Duox1 positive feedback inflammatory loop triggered in the skin of Tnfa- or Tnfr2-deficient zebrafish. Stage 1 (left panel): Tnfa or Tnfr2 deficiency triggers Duox1-dependent release of H_2_O_2_, which in turn promotes Lyn-mediated neutrophil infiltration. Stage 2 (right panel): H_2_O_2_ induces the activation of NF-κB, which is then translocated to the nucleus and induces the activation of genes encoding pro-inflammatory mediators (Il1b, Ptgs2, and probably Duox1). Pharmacological or genetic inhibition of Duox1 restores skin homeostasis.

Increased production of TNFα is associated with the development of autoimmune/chronic inflammatory diseases, including psoriasis, lichen planus, rheumatoid arthritis, and IBD. We have used the unique advantages of the zebrafish embryo for *in vivo* imaging and cell tracking to demonstrate that the genetic depletion of Tnfa or Tnfr2, but not Tnfr1, caused the infiltration of neutrophils into the skin and hyperproliferation of keratinocytes through the activation of an H_2_O_2_/NF-κB/Duox1 positive feedback inflammatory loop (Figure 8). Strikingly, neutrophils, but not macrophages, are rapidly attracted to the skin. However, the activation of NF-κB and the induction of the gene encoding Il1b in the skin occurred before the appearance of the first neutrophils in the developing embryo. More importantly, DUOX1 was also strongly induced in the skin lesions of psoriasis and lichen planus patients. Collectively, these results (i) indicate a critical role of TNFα/TNFR2 signaling in the protection of the skin against oxidative stress, (ii) might explain the appearance of psoriasis and lichen planus in patients treated with anti-TNFα therapies [5]–[10], and (iii) support the idea that specific inhibition of the TNFα/TNFR1 signaling axis while leaving TNFα/TNFR2 signaling unaffected would inhibit the pathological effects of TNFα and reduce the side effects associated with this therapy [19],[36]. This apparent discrepancy with TNFα-deficient mice, which do not show skin inflammation, may be due to developmental and/or physiological compensations, which probably do not exist in humans [37]–[39].

One of the most intriguing observations from this study is that impaired Tnfr2 signaling led to the induction of *duox1* and the production of H_2_O_2_ by keratinocytes. H_2_O_2_ gradient was recently shown to contribute to the early influx of neutrophils in wound [31] and tumor [32], although it seems to be dispensable for neutrophil detection of localized infection [33]. To the best of our knowledge, this is the first study showing a role for Duox1-derived H_2_O_2_ in the induction of NF-κB in the skin *in vivo*, suggesting that H_2_O_2_ might play a critical role in the initiation and maintenance of chronic inflammatory diseases in both zebrafish and human. These observations suggest that antioxidants or inhibition of Duox1 might be therapeutic for the treatment of patients suffering from psoriasis, lichen planus, and other inflammatory diseases. Supporting this notion, several studies using psoriasis and IBD mouse models have shown that transgenic overexpression of endogenous antioxidant genes promotes protection, while antioxidant gene knockout promotes sensitization (reviewed by [40],[41]). Even more importantly, the antioxidant levels and the oxidative stress biomarkers are usually correlated with the disease severity and the extent of inflammation in the psoriasis and IBD patients [40]–[42]. Therefore, all these results taken together suggest that antioxidants should be considered as part of a more specific and effective therapy for the treatment of inflammatory skin diseases, including psoriasis and lichen planus. The ability of Duox1 inhibition by pharmacological approaches, but not of IL-1β, to restore skin homeostasis in Tnfa- and Tnfr2-deficient zebrafish embryos further supports this conclusion.

It is known that different reactive oxygen species (ROS) act as second messengers, influencing various cellular signal transduction pathways, including NF-κB. However, there are still many inconsistencies concerning the influence of oxidative stress on NF-κB activity [43], and unfortunately, most studies have been performed *in vitro* using H_2_O_2_ and cultured cells [44],[45]. Such studies have shown that H_2_O_2_ can act as an activator of IκB kinases (IKKs) [46] or can inactivate these proteins [47], probably depending on the cell type. More recently, it has been found that the same prolyl hydroxylases that confer oxygen sensitivity to the hypoxia-inducible factor (HIF) pathway, namely PHD1 and PHD2, seem to act as repressors of the canonical NF-κB pathway through mechanisms that could include direct hydroxylation of IKKβ [48]. Our epistasis study in zebrafish demonstrates for the first time that the absence of Tnfa/Tnfr2 signaling led to the production of H_2_O_2_ by keratinocytes, which, in turn, resulted in NF-κB activation and the induction of genes encoding pro-inflammatory mediators. This self-perpetuating cycle may be of clinical importance in view of the presumably key role played by oxidative stress [40]–[42], HIF [49],[50], and NF-κB in psoriasis and IBD. It is tempting to speculate that the Tnfa/Tnfr2 axis would be required to prevent skin oxidative stress through the regulation of ROS-detoxifying enzymes, as it has been reported for oligodendrocyte progenitor cells *in vitro* [51]. The model reported here might contribute to clarify the mechanisms involved in the regulation of oxidative stress by TNFα, the regulation of NF-κB activity by ROS, and the crosstalk between oxidative stress and inflammation *in vivo*.

The essential role played by NF-κB in the homeostasis of the skin is evidenced by the human X-linked genodermatosis IP, which affects the regulatory subunit of IKK (IKKγ, NEMO) [17]. Humans suffering from this genetic disease exhibit severe skin inflammation, paradoxically due to impaired NF-κB activation and reduced resistance to TNFα/TNFR1-mediated apoptosis [52],[53]. Similarly, although NF-κB actively participates in the excessive inflammatory response observed in IBD patients [54],[55], recent studies with mice defective in NF-κB activation have revealed that epithelial NF-κB activation is essential to preserve intestinal homeostasis [56],[57]. Therefore, a critical NF-κB signaling balance is required for skin and gut homeostasis, as both excessive and defective epithelial NF-κB activation can result in inflammation. Similarly, although the TNFα/TNFR1 axis was earlier appreciated to be involved in the apoptosis of both keratinocytes and enterocytes in the absence of NF-κB signaling [52],[53],[56],[57], our results show that TNFα signaling through TNFR2 is also critically required for skin homeostasis. Whether the TNFα/TNFR2 axis is also required for gut homeostasis will require further investigation using germ-free and gnotobiotic zebrafish larvae, as host–microbe interactions have a profound impact in gut physiology and are usually involved in IBD.

In conclusion, we have found that Tnfa signaling through Tnfr2 is indispensably required for the protection of the skin against oxidative stress-induced inflammation in the zebrafish. Thus, the absence of this signal triggers the local production of H_2_O_2_ by Duox1, which, in turn, activates NF-κB and results in the up-regulation of genes encoding pro-inflammatory mediators and neutrophil infiltration. These results, together with the induction of DUOX1 in the skin lesions of psoriasis and lichen planus patients, reveal a crucial role of H_2_O_2_ and DUOX1 in skin inflammation and suggest that pharmacologic and genetic therapies that target these two key factors could provide innovative approaches to the management of psoriasis, lichen planus, and other chronic inflammatory diseases.

## Materials and Methods

### Ethics Statement

The experiments performed comply with the Guidelines of the European Union Council (86/609/EU). Experiments and procedures were performed as approved by the Bioethical Committee of the University of Murcia (approval no. 537/2011) and the Ethical Clinical Research Committee of the University Hospital Virgen de la Arrixaca (approval no. 8/13).

### Animals

Zebrafish (*Danio rerio* H.) were obtained from the Zebrafish International Resource Center and mated, staged, raised, and processed as described [58]. The lines *Tg(mpx:eGFP)^i114^* [59], Tg(*lyz:dsRED*)^nz50^ [60], Tg(*mpeg1:eGFP*)^gl22^ [61], and *Tg(krt18:RFP)* [62] were previously described. The *Tg*(*NFκB-RE*:eGFP) (*NF-κB:eGFP* for simplicity) line was generated with the method and constructs previously described [28].

### MO, RNA Injection, and Chemical Treatments

Specific MOs (Gene Tools) were resuspended in nuclease-free water to 1 mM (Table S1). *In vitro*–transcribed RNA was obtained following the manufacturer's instructions (mMESSAGE mMACHINE Kit, Ambion). MOs and RNA (200 pg/egg) were mixed in microinjection buffer (0.5× Tango buffer and 0.05% phenol red solution) and microinjected into the yolk sac of one- to eight-cell-stage embryos using a microinjector (Narishige) (0.5–1 nl per embryo). The same amounts of MOs and/or RNA were used in all experimental groups. The efficiency of the MOS was checked by RT-PCR as described previously [19],[27],[31],[34].

In some experiments, 1 dpf embryos were manually dechorionated and/or treated for 24 h at 28°C by bath immersion with the NADPH oxidase inhibitor dibenziodolium chloride (DPI, Sigma-Aldrich) at a final concentration of 100 µM diluted in egg water supplemented with 1% DMSO.

### Live Imaging of Zebrafish Larvae

At 72 hpf, larvae were anesthetized in tricaine and mounted in 1% (wt/vol) low-melting-point agarose (Sigma-Aldrich) dissolved in egg water [63]. Images were captured with an epifluorescence Lumar V12 stereomicroscope equipped with green and red fluorescent filters while animals were kept in their agar matrixes at 28.5°C. All images were acquired with the integrated camera on the stereomicroscope and were used for subsequently counting the number of neutrophils (*mpx:eGFP*) and examining their distribution. The activation of NF-κB was visualized and quantified using the line *NF-κB*::eGFP. Stacked images were captured using 1 µm (neutrophil infiltration into the skin) or 25 µm (neutrophil distribution, NF-κB activation, and H_2_O_2_ formation) increments and deconvolved using Huygens Essential Confocal software (v 4.1 0p6b) by Scientific Volume Imaging. Stacks were processed using the free source software ImageJ (http://rsbweb.nih.gov/ij) to obtain a maximum intensity projection of the *xy* axis of the stack. For the quantification of neutrophil distribution and NF-κB activation, the maximum projection for each larva was then converted to a fluorescence value matrix, where the value obtained for each pixel transversally was the mean ± S.E.M. for all the pixels for each row (15 larvae per treatment from 3 different experiments). In parallel, the activation of NF-κB in the skin was also quantified by the skin NF-κB activation index, which was defined as the fluorescence in the skin (a+b) relative to the total fluorescence of the larvae (c).

H_2_O_2_ imaging using a live cell fluorogenic substrate was performed essentially as previously described [32]. Briefly, 3-dpf tnfα and Tnfr2 morphants and their control siblings were loaded for 30 min with 50 µM acetyl-pentafluorobenzene sulphonyl fluorescein (Cayman Chemical) in 1% DMSO in egg water and imaged as above. As a positive control, complete transfection of the tail of anesthetized 72 hpf larvae was performed with a disposable sterile scalpel [63].

### Flow Cytometry

At 3 dpf, approximately 300 to 500 *Tg(mpx:eGFP) and Tg(krt18:RFP)* larvae were anesthetized in tricaine, minced with a razor blade, incubated at 28°C for 30 min with 0.077 mg/ml Liberase (Roche), and the resulting cell suspension passed through a 40 µm cell strainer. Sytox (Life Technologies) was used as a vital dye to exclude dead cells. Flow cytometric acquisitions were performed on a FACSCALIBUR (BD), and cell sorting was performed on a Coulter (Epics Altra). Analyses were performed using FlowJo software (Treestar).

### Analysis of Gene Expression

Total RNA was extracted from whole embryos/larvae or sorted cell suspensions with TRIzol reagent (Invitrogen) following the manufacturer's instructions and treated with DNase I, amplification grade (1 U/µg RNA; Invitrogen). SuperScript III RNase H^−^ Reverse Transcriptase (Invitrogen) was used to synthesize first-strand cDNA with oligo(dT)18 primer from 1 µg of total RNA at 50°C for 50 min. Real-time PCR was performed with an ABI PRISM 7500 instrument (Applied Biosystems) using SYBR Green PCR Core Reagents (Applied Biosystems). Reaction mixtures were incubated for 10 min at 95°C, followed by 40 cycles of 15 s at 95°C, 1 min at 60°C, and finally 15 s at 95°C, 1 min 60°C, and 15 s at 95°C. For each mRNA, gene expression was normalized to the ribosomal protein S11 (*rps11*) content in each sample using the Pfaffl method [64]. The primers used are shown in Table S2. In all cases, each PCR was performed with triplicate samples and repeated at least with two independent samples.

### Histology and WIHC

Larvae were fixed overnight in 4% paraformaldehyde solution (PFA), embedded in Paraplast Plus (Sherwood Medical), and sectioned at a thickness of 5 µm. After being dewaxed and rehydrated, they were stained with haematoxylin and eosin (H&E).

Tg(*mpx:eGFP*) or Tg(NF-κB:eGFP); *Tg(krt18:RFP)* 3 dpf larvae were fixed overnight at 4°C in 4% PFA at room temperature, dehydrated in methanol/PBS solutions (25, 50, 75, and 100%, 5 min each), and stored in 100% methanol at −20°C. For staining, larvae were rehydrated in 75, 50, and 25% methanol/PBT (PBS and 0.1% Tween-20) solutions for 5 min each, washed three times for 5 min in PBT, incubated for 5 min RT with 150 mM Tris-HCl pH 9, followed by heating at 70°C for 15 min [65]. After the heating treatment, larvae were directly washed twice in PBT for 10 min and twice in dH_2_O for 5 min. Subsequently, to enhance tissue permeabilization, larvae were incubated with cold acetone for 20 min at −20°C, washed twice in dH_2_O and twice in PBT (5 min each), followed by blocking with blocking solution (PDT = PBT+1% DMSO) supplemented with 5% FBS and 2 mg/ml BSA) for 2 h at 22°C. After blocking, embryos were incubated overnight at 4°C with primary antibodies diluted (1∶200) in blocking buffer, washed three times in PDT (15 min each), and blocked again for 2 h at 22°C. Secondary antibody staining was done for 2 h RT at 1∶500 dilution in blocking buffer, and larvae were then washed five times in PBT (5 min each) and stored in Vectashield (Vector Labs) until image acquisition. The following primary antibodies were used: rabbit anti-phosphorylated-Histone H3 (Ser 10)-R (#SC8656-R, Santa Cruz Biotechnology) and rabbit anti-human p63 (#SC8343, Santa Cruz Biotechnology). Mouse anti-RFP (#MA5-15257, Thermo Scientific) and Alexa Fluor 594 (#A11032) and Alexa Fluor 532 (#A11002) Goat Anti-Mouse IgG (H+L) (Life Technologies) were used as secondary antibodies.

Confocal immunofluorescence images were acquired with a confocal microscope (LEICA TCS-SP2, Leica) using an NA 0.70/20× dry objective. Z-series were acquired using a 210–300 µm pinhole. The 2D and 3D maximum intensity projections and corresponding animation videos were made using ImageJ (http://rsb.info.nih.gov/ij/).

### Human Skin Samples

Skin biopsies from healthy donors (*n* = 10) and lichen planus (*n* = 8) and psoriasis patients (*n* = 15) were fixed in 4% PFA, embedded in Paraplast Plus, and sectioned at a thickness of 5 µm. After being dewaxed and rehydrated, the sections were incubated in 50 mM glycine-HCl buffer (pH 3.5) containing 0.01% ethylenediaminetetraacetic acid (EDTA) at 95°C for 5 min and then at room temperature for 20 min to retrieve the antigen. Afterwards, they were immunostained with a 1/50 dilution of a goat polyclonal antibody to human DUOX1 (sc-48858, Santa Cruz Biotechnology) followed by ImmunoCruz goat ABC Staining System (sc-2023, Santa Cruz Biotechnology) following the manufacturer's recommendations. The specificity of the staining was confirmed by pre-incubating a 10-fold excess (in molarity) of a commercial blocking peptide (sc-48858 P, Santa Cruz Biotechnology) with the DUOX1 antibody overnight at 4°C. No staining was observed in these conditions. Sections were finally examined under a Leica microscope equipped with a digital camera Leica DFC 280, and the photographs were processed with Leica QWin Pro software.

### Statistical Analysis

Data were analyzed by analysis of variance (ANOVA) and a Tukey multiple range test to determine differences between groups. The differences between two samples were analyzed by the Student *t* test. The contingency graphs were analyzed by the Chi-square (and Fisher's exact) test.

## Supporting Information

Figure S1 Tnfa and Tnfr2 deficiencies result in neutrophil mobilization. Zebrafish one-cell *mpx:eGFP* embryos were injected with standard control (Std), Tnfr1, Tnfr2, Tnfa, or Tnfr1+Tnfr2 MOs alone or combination with antisense (As), Tnfa, Tnfr2, or DN-Tnfr2 mRNAs. The phenotype of 3 dpf larvae was classified as neutrophil grouped in the CHT or scattered, as described in Figure 1. ***p<0.001.
(TIF)

Figure S2 Macrophage distribution is not altered in Tnfa- or Tnfr2-deficient larvae. Zebrafish one-cell *mpeg1:eGFP* embryos were injected with standard control (Std), Tnfr2, and Tnfa MOs. Representative images showing macrophage distribution in 72 hpf larvae. Scale bars, 100 µm.
(TIF)

Figure S3 Efficiency of sorting of neutrophils and keratinocytes. Zebrafish one-cell *mpx*:eGFP (A) or *krt18*:RFP (B) embryos were injected with standard control (Std) or Tnfr2 MOs. Neutrophils (A) and keratinocytes (B) were FACS-sorted from 72 hpf larvae, and the expression of *gfp* and *mpx* (A) and *krt18* and *p63* (B) genes was measured by RT-qPCR in unsorted and sorted cells. The data are shown as the mean ± S.E.M. ns, not significant. *p<0.05; ***p<0.001.
(TIF)

Figure S4 Neutrophils and keratinocytes expressed both Tnf receptors. Neutrophils (A) and keratinocytes (B) were FACS-sorted from 72 hpf *mpx:eGFP* and *krt18:RFP* larvae, respectively, and the expression of *tnfr1* and *tnfr2* genes was measured by RT-qPCR in unsorted and sorted cells. The data are shown as the mean ± S.E.M. ns, not significant. **p<0.01; ***p<0.001.
(TIF)

Figure S5 IL-1β is induced in Tnfr2-deficient embryos before the emergence of neutrophils. Zebrafish one-cell wild-type embryos were injected with standard control (Std) or Tnfr2 MOs. The expression of *il1b* gene was measured by RT-qPCR in whole embryos at 24 and 48 hpf. The data are shown as the mean ± S.E.M. *p<0.05.
(TIF)

Figure S6 The skin of Tnfr2-deficient larvae does not show histopathological alterations. Zebrafish one-cell embryos were injected with standard control (Std) or Tnfr2 MOs. At 3 (A) and 5 (B) dpf, the larvae were fixed, embedded in Paraplast Plus, sectioned at 5 µm, and stained with H&E. M, muscle. Arrowheads, skin. Scale bars, 50 µm.
(TIF)

Figure S7 Pharmacological inhibition of Duox1 inhibits H_2_O_2_ production after wounding. Zebrafish one-cell wild-type embryos were treated at 72 hpf by immersion in 100 µM DPI or vehicle alone (DMSO) in the presence of 50 µM acetyl-pentafluorobenzene sulphonyl fluorescein, and tailfins were then transected. Representative images of the formation of the H_2_O_2_ gradient at 1 h postwounding. Note that DPI treatment completely inhibits H_2_O_2_ formation at the wound. Scale bars, 100 µm.
(TIF)

Figure S8 H_2_O_2_ production by Tnfr2-deficient keratinocytes preceded the activation of NF-κB. Zebrafish one-cell wild-type (A, B) or *lyz:dsRED*; *NF-κB:eGFP* (C, D) embryos were injected with standard control (Std) or Tnfr2 MOs. (A, B) Larvae were dechorionated at 24 hpf and then labeled with 50 µM acetyl-pentafluorobenzene sulphonyl fluorescein at 24, 48, and 72 hpf. Representative images of green channels of Std and Tnfr2 morphants (A) and quantification of green fluorescence in the indicated number of larvae (B) are shown. Note that increased H_2_O_2_ production by skin keratinocytes is already observed at 24 hpf. (C) Representative pictures showing NF-κB activation levels in control and Tnfr2-deficient larvae at 24, 48, and 72 hpf. Note that NF-κB is induced in the skin (red arrowheads) of Tnfr2-deficient larvae at 48 h and that neutrophil dispersion is observed at 72 hpf and, to some extent, at 48 hpf. The neuromasts are indicated with white arrowheads. (D) Quantification of the percentage of larvae showing activation of the NF-κB in the skin. The results are shown as the mean ± S.E.M. The number of larvae analyzed is also indicated. Scale bars, 100 µm. ns, not significant; auf, arbitrary units of fluorescence. *p<0.05; **p<0.01; ***p<0.001.
(TIF)

Figure S9 Genetic inactivation of Duox1 using a DN form partially prevents neutrophil infiltration into the skin of Tnfa- and Tnfr2-deficient zebrafish. Zebrafish one-cell *mpx:eGFP* embryos were injected with standard control (Std), Tnfr2, or Tnfa MOs alone or combination with antisense (As) or DN-Duox1 mRNAs. Representative images of bright field and green channels of morphants at 72 hpf showing the differences in the neutrophils distribution (A) and quantification of neutrophil mobilization from the CHT to the skin in the indicated number of larvae per group from three different experiments (B). The mean ± S.E.M. for each group is shown. Scale bars, 100 µm. ns, not significant. ***p<0.001.
(TIF)

Table S1 MOs used in this study. The gene symbols followed the Zebrafish Nomenclature Guidelines (http://zfin.org/zf_info/nomen.html). ENA, European Nucleotide Archive (http://www.ebi.ac.uk/ena/).
(DOCX)

Table S2 Primers used in this study. The gene symbols followed the Zebrafish Nomenclature Guidelines (http://zfin.org/zf_info/nomen.html). ENA, European Nucleotide Archive (http://www.ebi.ac.uk/ena/).
(DOCX)

Video S1 Neutrophils infiltrate the skin in Tnfr2-deficient larvae. Animations of tridimensional projections obtained by laser confocal microscopy of STD morphants showing neutrophils in green (GFP) and basal keratinocytes in red (p63). See legend to Figure 1 for details.
(AVI)

Video S2 Neutrophils infiltrate the skin in Tnfr2-deficient larvae. Animations of tridimensional projections obtained by laser confocal microscopy of Tnfr2 morphants showing neutrophils in green (GFP) and basal keratinocytes in red (p63). See legend to Figure 1 for details.
(AVI)

Video S3 NF-κB is induced in the skin of Tnfr2-deficient larvae. Animations of tridimensional projections obtained by laser confocal microscopy of STD morphants showing NF-κB activity in green (GFP) and basal keratinocytes in blue (RFP). See legend to Figure 3 for details.
(AVI)

Video S4 NF-κB is induced in the skin of Tnfr2-deficient larvae. Animations of tridimensional projections obtained by laser confocal microscopy of Tnfr2 morphants showing NF-κB activity in green (GFP) and basal keratinocytes in blue (RFP). See legend to Figure 3 for details.
(AVI)

## Abbreviations

CHT: caudal hematopoietic tissue
DN: dominant negative
Duox: dual oxidase
HIF, hypoxia-inducible factor; H&E, haematoxylin and eosin; IBD: inflammatory bowel disease
Il1b: interleukin-1β
IP: incontinentia pigmenti
MO: morpholino
PFA, paraformaldehyde; Ptgs2: prostaglandin-endoperoxide synthase 2
ROS: reactive oxygen species
Tnf: tumor necrosis factor, Tnfr, Tnf receptor
