# Supplementary figures and images for: Tnfa Signaling Through Tnfr2 Protects Skin Against Oxidative Stress–Induced Inflammation
Source: PLoS Biol. 2014 May 6;12(5):e1001855. doi: 10.1371/journal.pbio.1001855 (PMC4011677; doi:10.1371/journal.pbio.1001855)

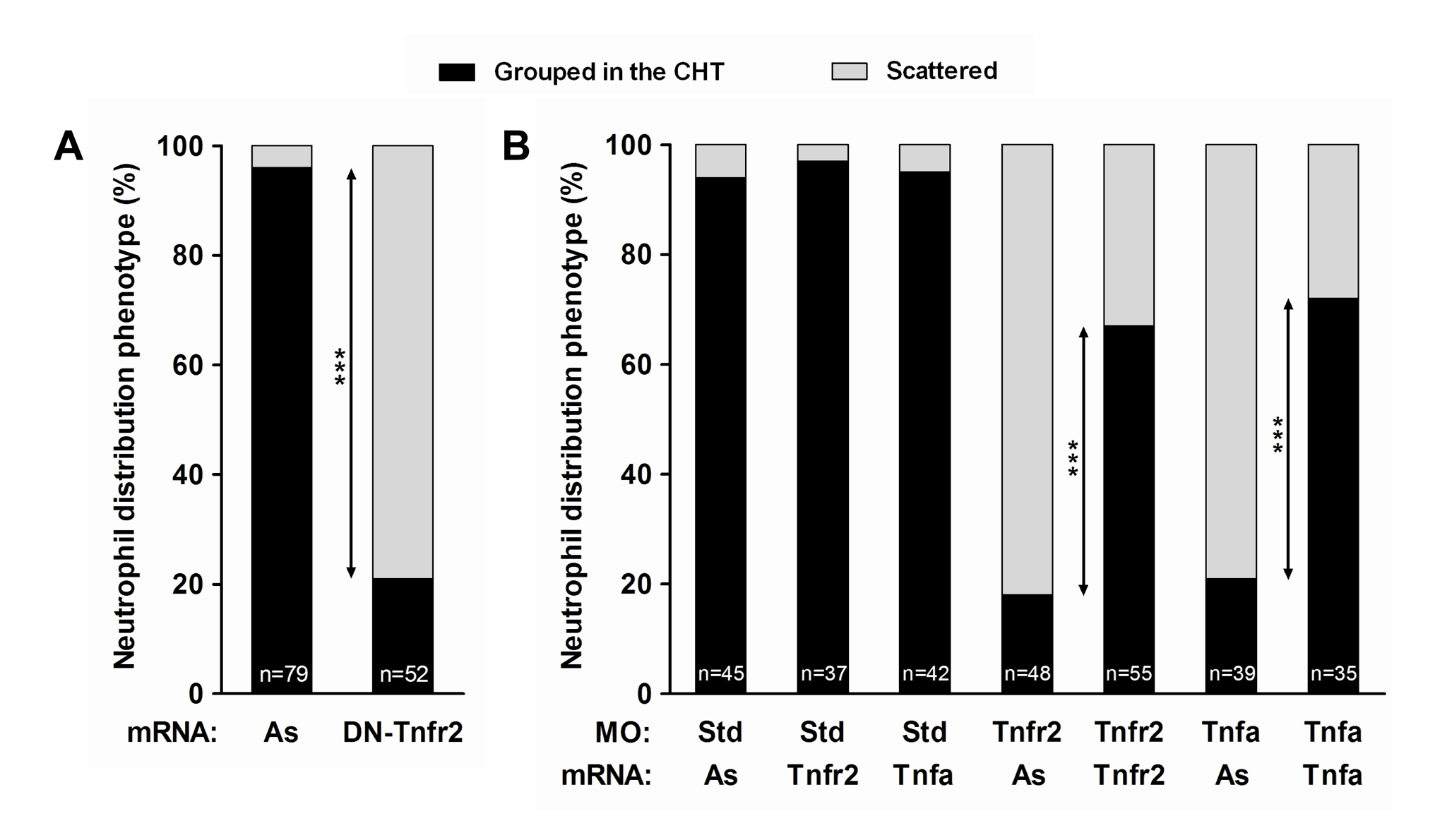

Supplement: Figure S1 — Tnfa and Tnfr2 deficiencies result in neutrophil mobilization. Zebrafish one-cell mpx:eGFP embryos were injected with standard control (Std), Tnfr1, Tnfr2, Tnfa, or Tnfr1+Tnfr2 MOs alone or combination with antisense (As), Tnfa, Tnfr2, or DN-Tnfr2 mRNAs. The phenotype of 3 dpf larvae was classified as neutrophil grouped in the CHT or scattered, as described in Figure 1. ***p<0.001. (TIF) [file pbio.1001855.s001.tif]

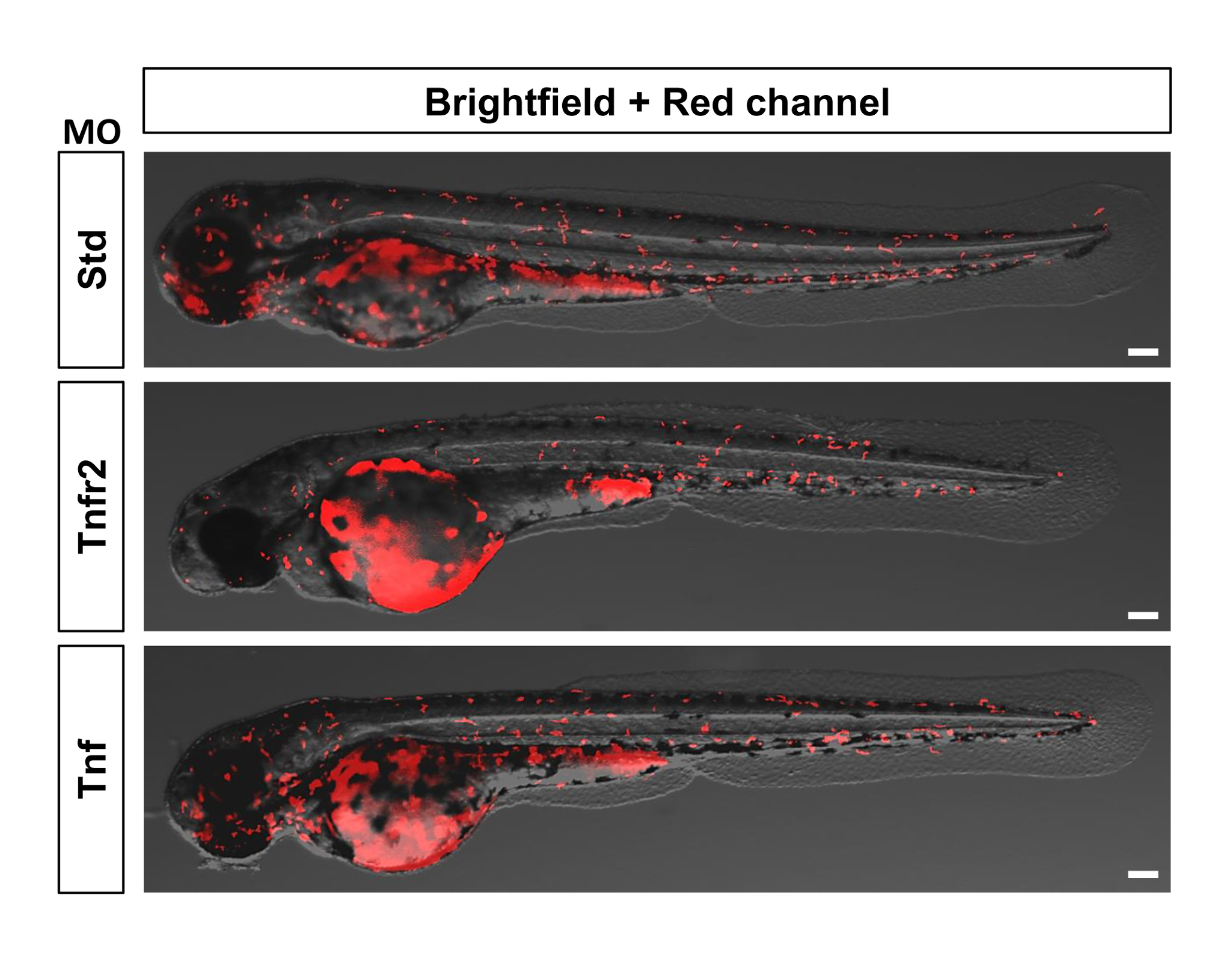

Supplement: Figure S2 — Macrophage distribution is not altered in Tnfa- or Tnfr2-deficient larvae. Zebrafish one-cell mpeg1:eGFP embryos were injected with standard control (Std), Tnfr2, and Tnfa MOs. Representative images showing macrophage distribution in 72 hpf larvae. Scale bars, 100 µm. (TIF) [file pbio.1001855.s002.tif]

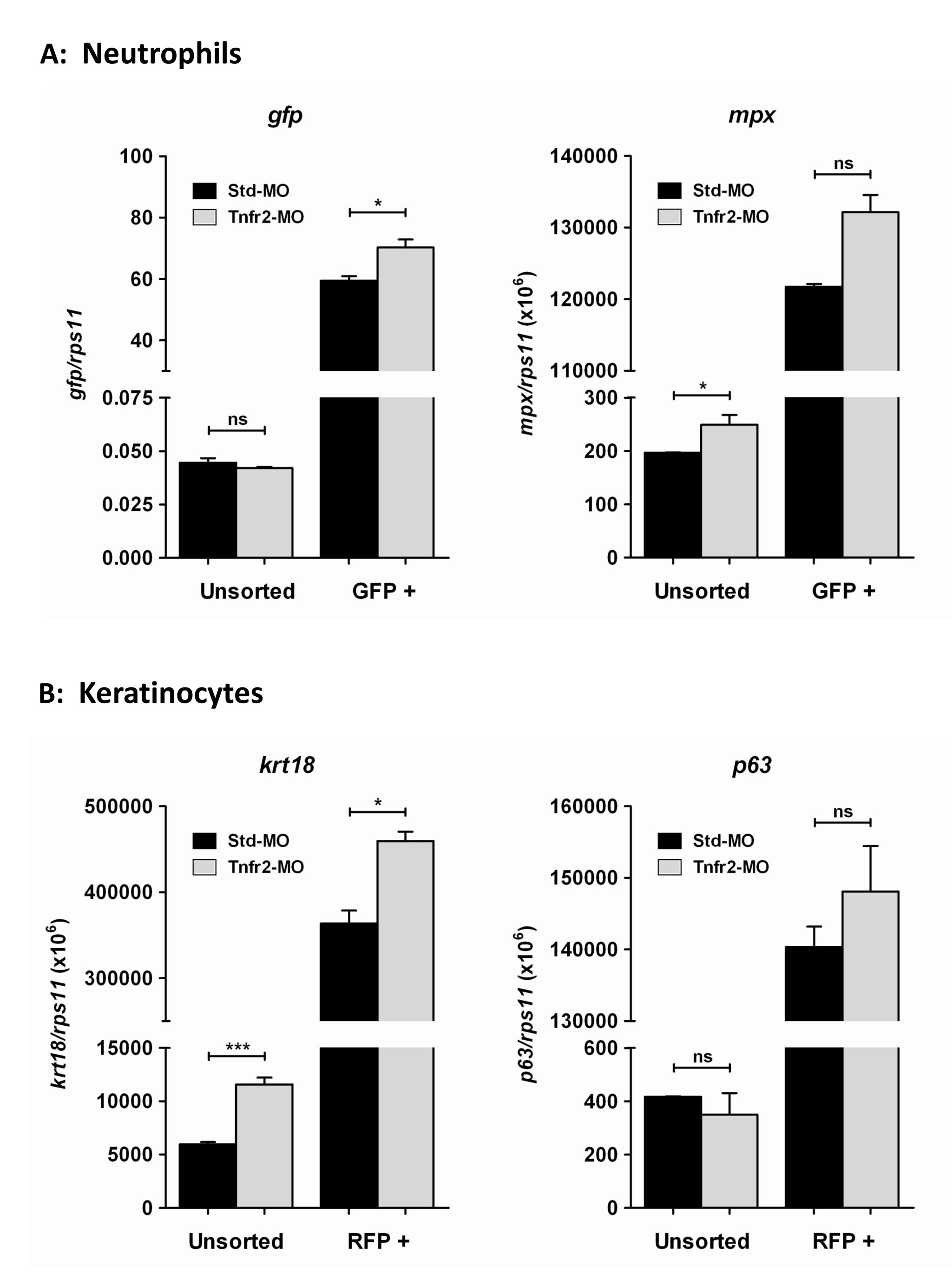

Supplement: Figure S3 — Efficiency of sorting of neutrophils and keratinocytes. Zebrafish one-cell mpx:eGFP (A) or krt18:RFP (B) embryos were injected with standard control (Std) or Tnfr2 MOs. Neutrophils (A) and keratinocytes (B) were FACS-sorted from 72 hpf larvae, and the expression of gfp and mpx (A) and krt18 and p63 (B) genes was measured by RT-qPCR in unsorted and sorted cells. The data are shown as the mean ± S.E.M. ns, not significant. *p<0.05; ***p<0.001. (TIF) [file pbio.1001855.s003.tif]

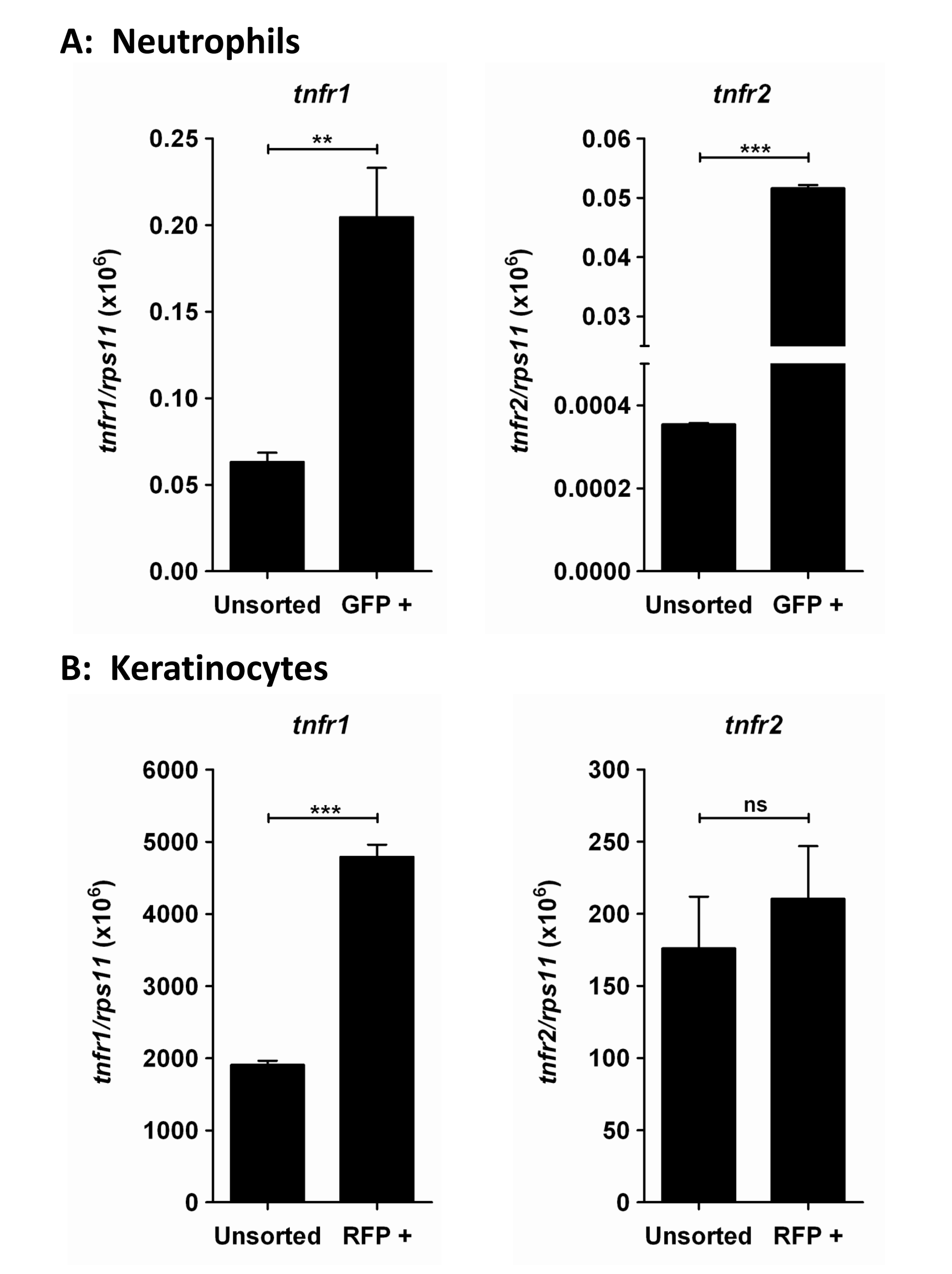

Supplement: Figure S4 — Neutrophils and keratinocytes expressed both Tnf receptors. Neutrophils (A) and keratinocytes (B) were FACS-sorted from 72 hpf mpx:eGFP and krt18:RFP larvae, respectively, and the expression of tnfr1 and tnfr2 genes was measured by RT-qPCR in unsorted and sorted cells. The data are shown as the mean ± S.E.M. ns, not significant. **p<0.01; ***p<0.001. (TIF) [file pbio.1001855.s004.tif]

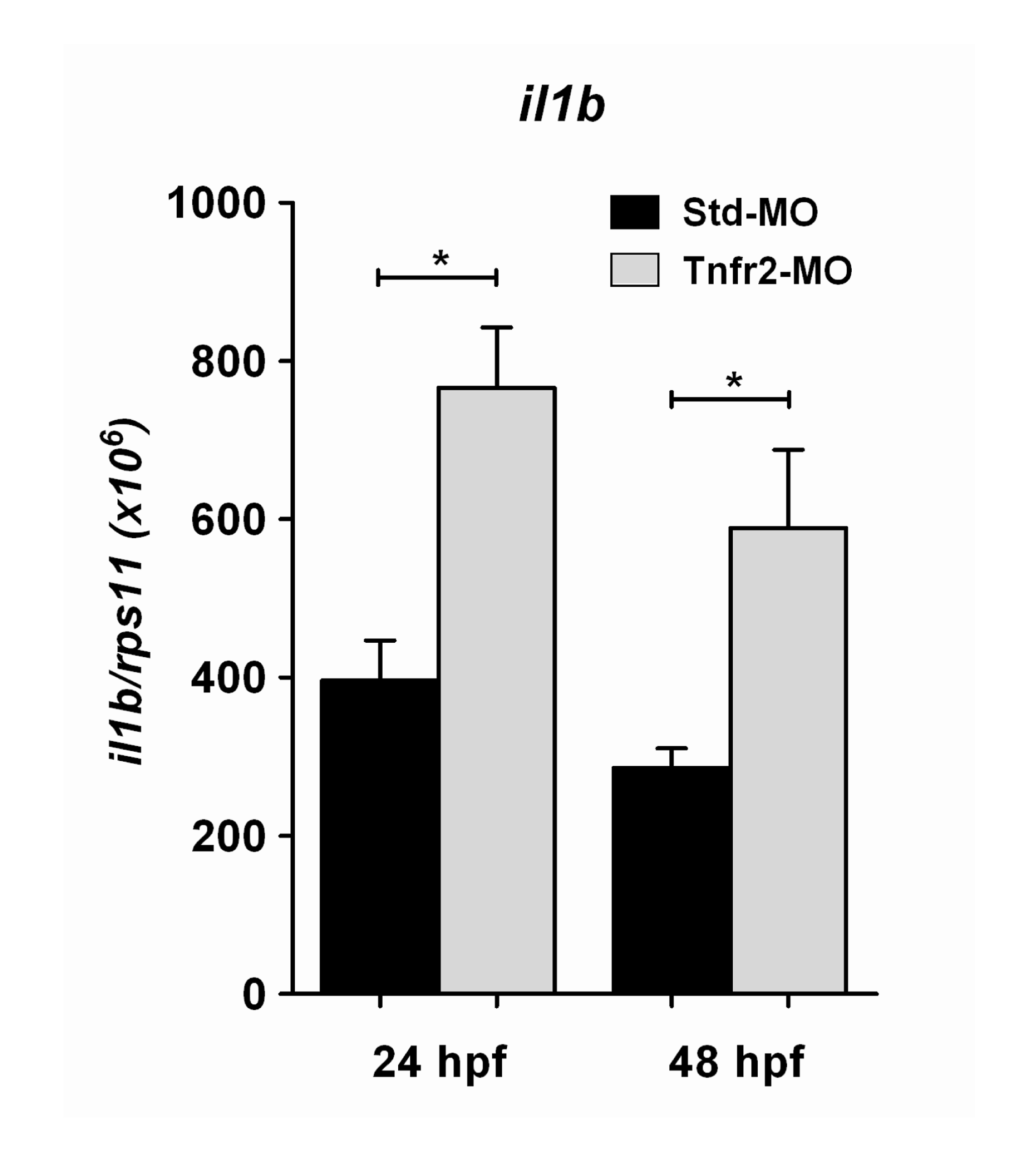

Supplement: Figure S5 — IL-1β is induced in Tnfr2-deficient embryos before the emergence of neutrophils. Zebrafish one-cell wild-type embryos were injected with standard control (Std) or Tnfr2 MOs. The expression of il1b gene was measured by RT-qPCR in whole embryos at 24 and 48 hpf. The data are shown as the mean ± S.E.M. *p<0.05. (TIF) [file pbio.1001855.s005.tif]

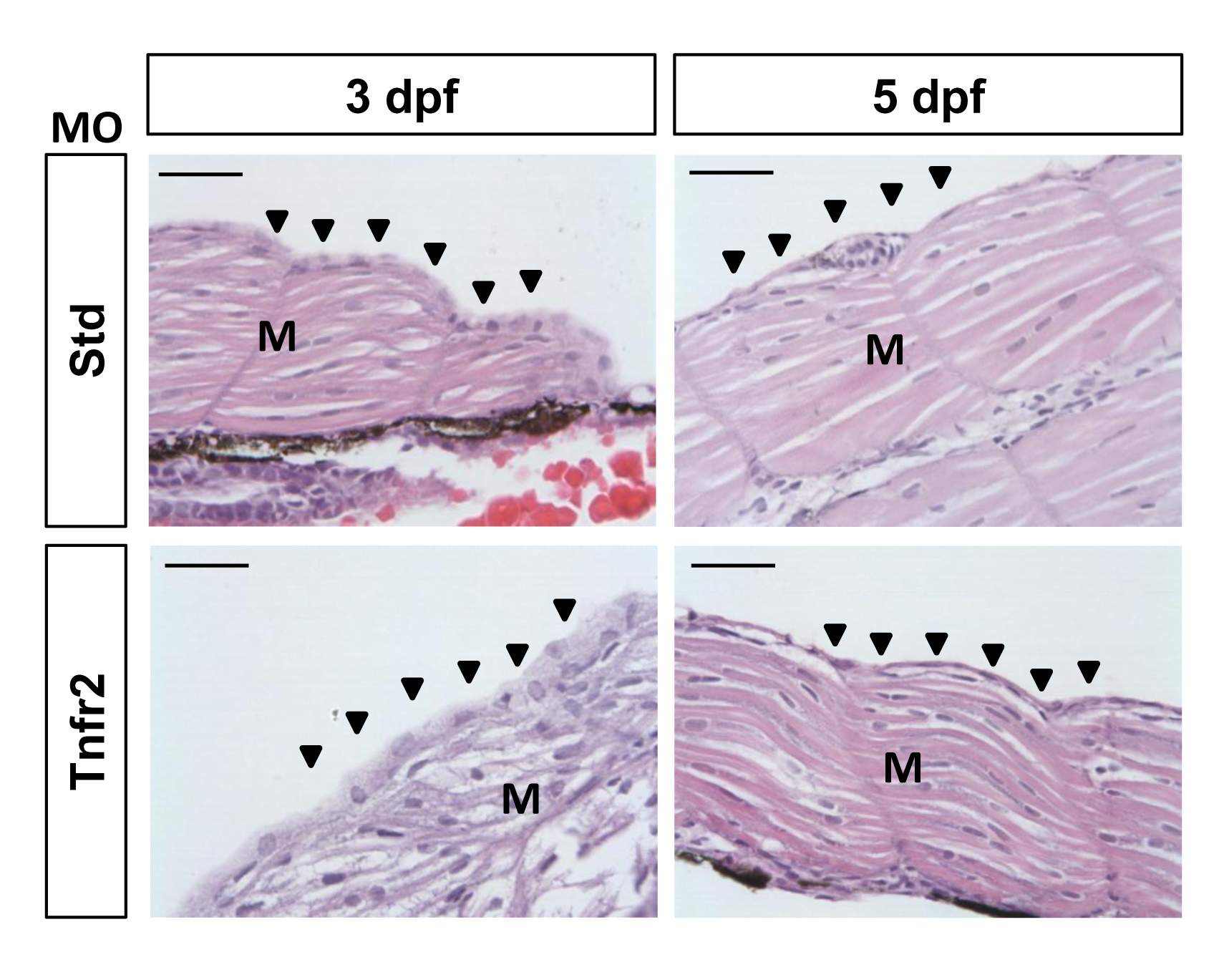

Supplement: Figure S6 — The skin of Tnfr2-deficient larvae does not show histopathological alterations. Zebrafish one-cell embryos were injected with standard control (Std) or Tnfr2 MOs. At 3 (A) and 5 (B) dpf, the larvae were fixed, embedded in Paraplast Plus, sectioned at 5 µm, and stained with H&E. M, muscle. Arrowheads, skin. Scale bars, 50 µm. (TIF) [file pbio.1001855.s006.tif]

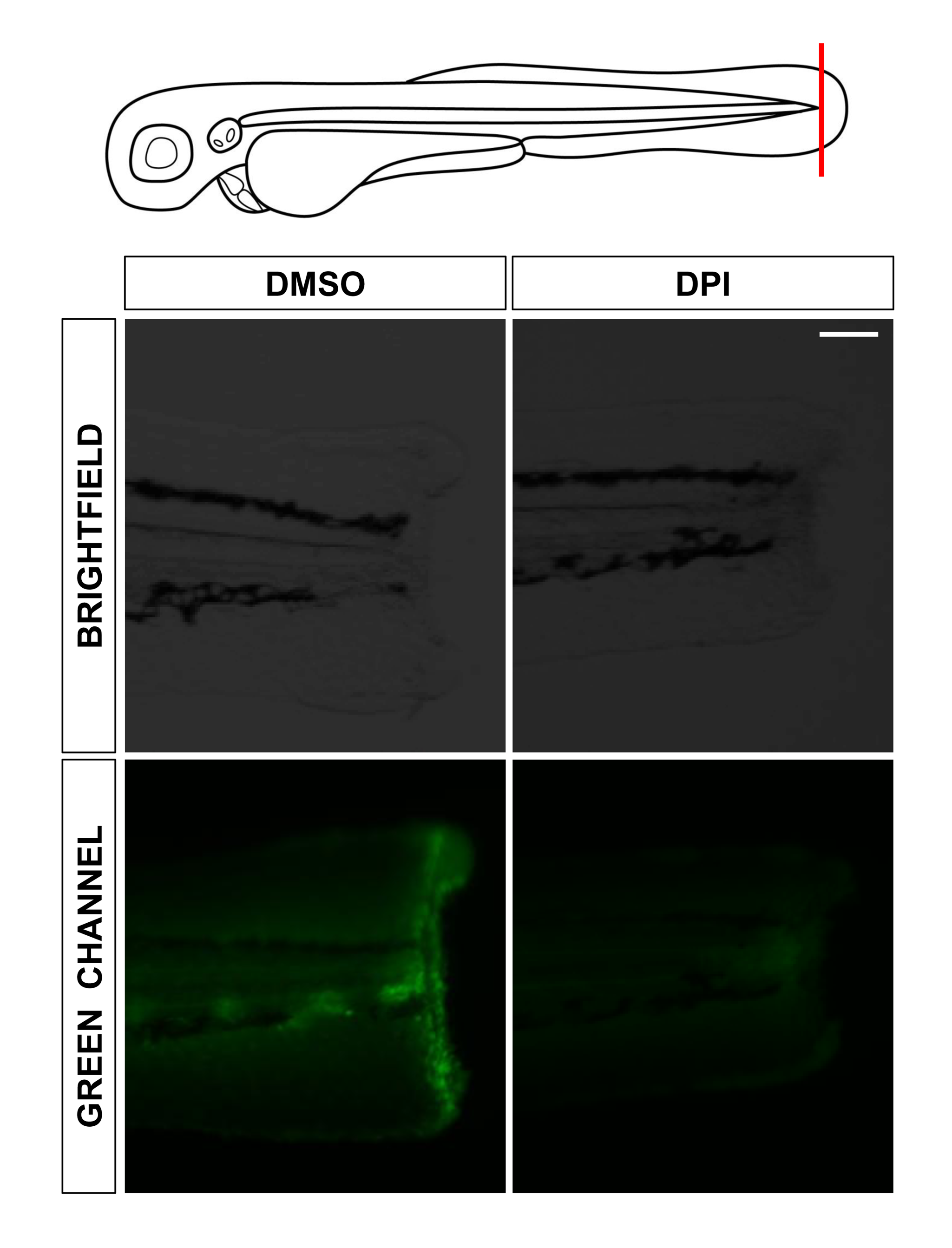

Supplement: Figure S7 — Pharmacological inhibition of Duox1 inhibits H2O2 production after wounding. Zebrafish one-cell wild-type embryos were treated at 72 hpf by immersion in 100 µM DPI or vehicle alone (DMSO) in the presence of 50 µM acetyl-pentafluorobenzene sulphonyl fluorescein, and tailfins were then transected. Representative images of the formation of the H2O2 gradient at 1 h postwounding. Note that DPI treatment completely inhibits H2O2 formation at the wound. Scale bars, 100 µm. (TIF) [file pbio.1001855.s007.tif]

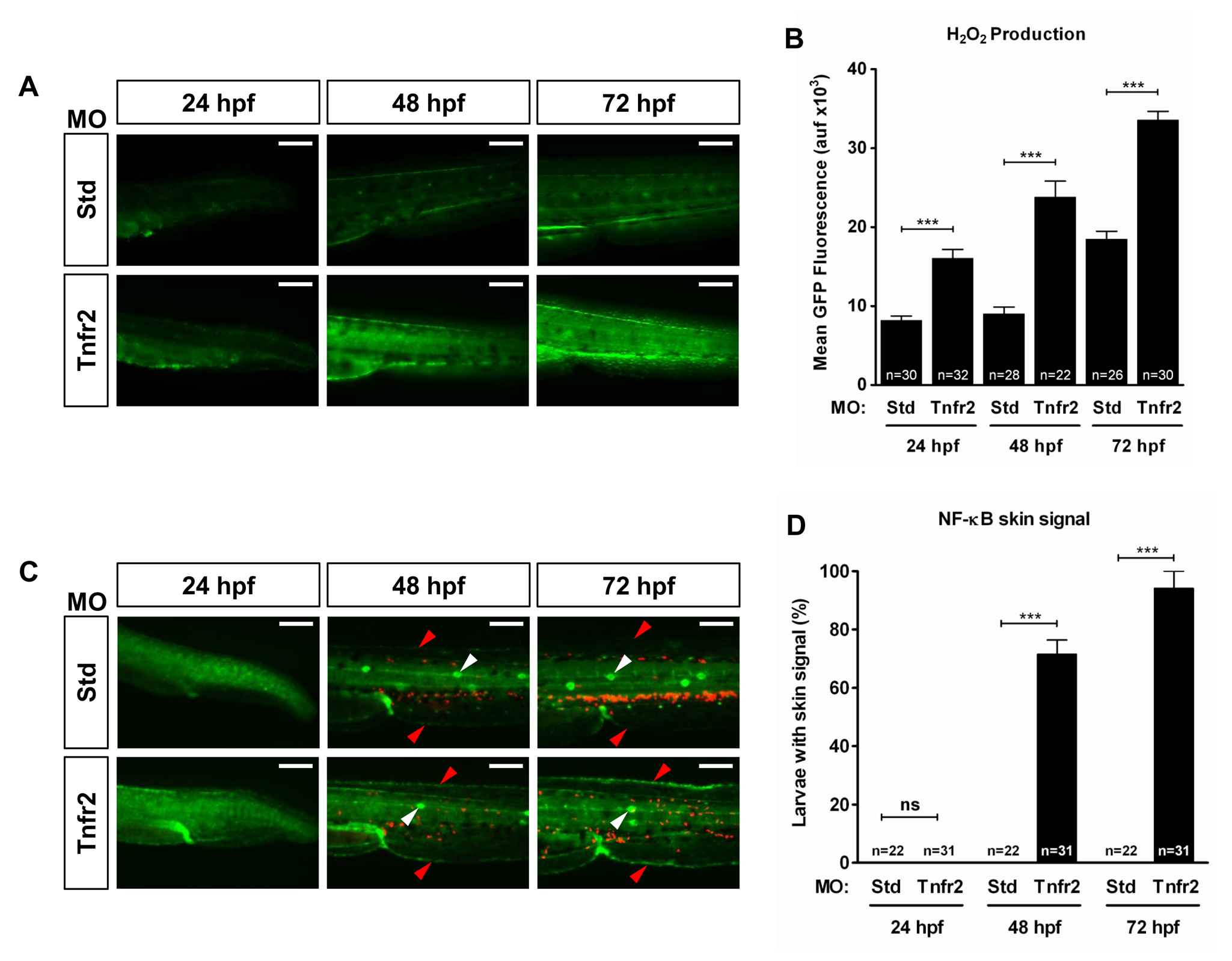

Supplement: Figure S8 — H2O2 production by Tnfr2-deficient keratinocytes preceded the activation of NF-κB. Zebrafish one-cell wild-type (A, B) or lyz:dsRED; NF-κB:eGFP (C, D) embryos were injected with standard control (Std) or Tnfr2 MOs. (A, B) Larvae were dechorionated at 24 hpf and then labeled with 50 µM acetyl-pentafluorobenzene sulphonyl fluorescein at 24, 48, and 72 hpf. Representative images of green channels of Std and Tnfr2 morphants (A) and quantification of green fluorescence in the indicated number of larvae (B) are shown. Note that increased H2O2 production by skin keratinocytes is already observed at 24 hpf. (C) Representative pictures showing NF-κB activation levels in control and Tnfr2-deficient larvae at 24, 48, and 72 hpf. Note that NF-κB is induced in the skin (red arrowheads) of Tnfr2-deficient larvae at 48 h and that neutrophil dispersion is observed at 72 hpf and, to some extent, at 48 hpf. The neuromasts are indicated with white arrowheads. (D) Quantification of the percentage of larvae showing activation of the NF-κB in the skin. The results are shown as the mean ± S.E.M. The number of larvae analyzed is also indicated. Scale bars, 100 µm. ns, not significant; auf, arbitrary units of fluorescence. *p<0.05; **p<0.01; ***p<0.001. (TIF) [file pbio.1001855.s008.tif]

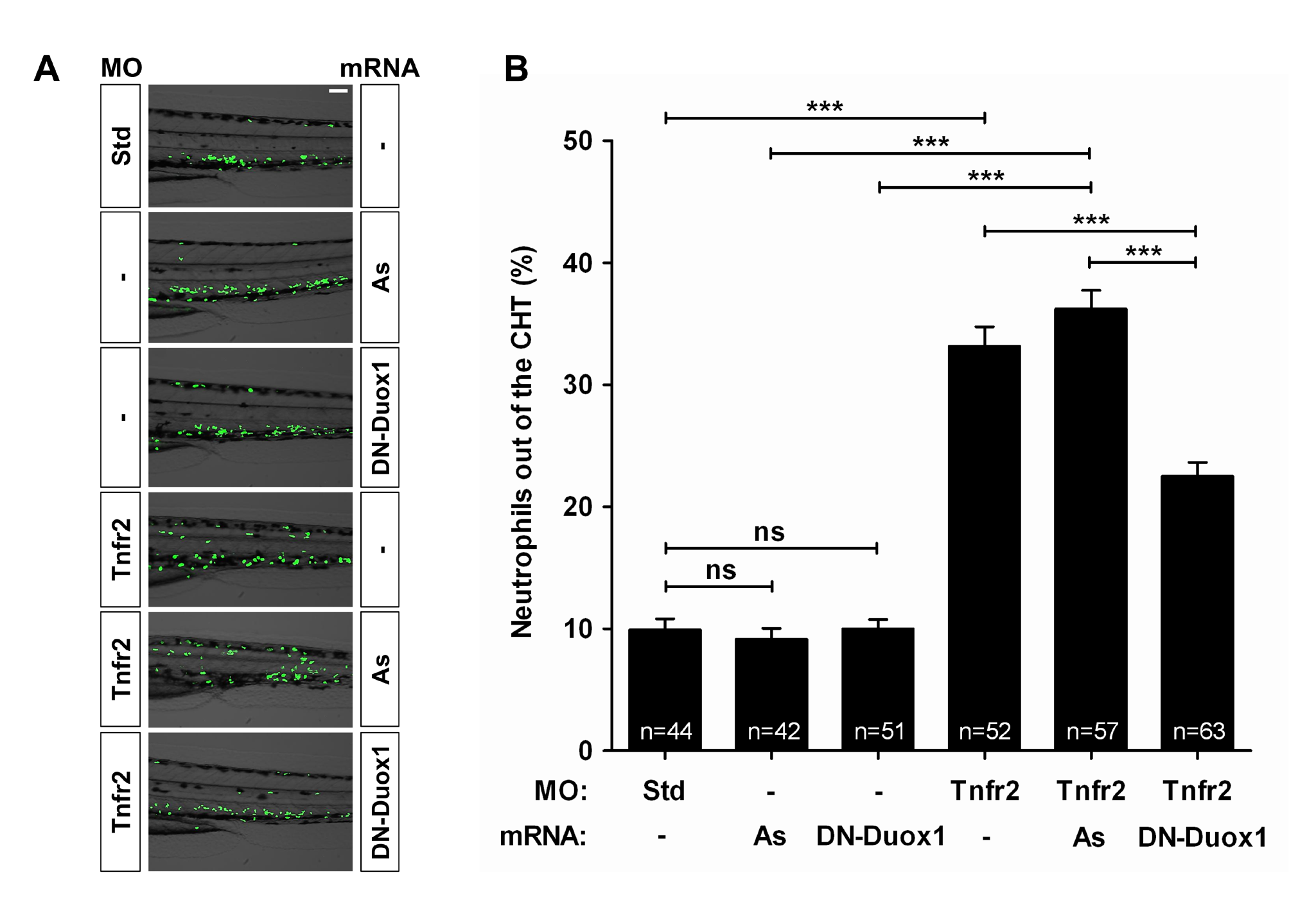

Supplement: Figure S9 — Genetic inactivation of Duox1 using a DN form partially prevents neutrophil infiltration into the skin of Tnfa- and Tnfr2-deficient zebrafish. Zebrafish one-cell mpx:eGFP embryos were injected with standard control (Std), Tnfr2, or Tnfa MOs alone or combination with antisense (As) or DN-Duox1 mRNAs. Representative images of bright field and green channels of morphants at 72 hpf showing the differences in the neutrophils distribution (A) and quantification of neutrophil mobilization from the CHT to the skin in the indicated number of larvae per group from three different experiments (B). The mean ± S.E.M. for each group is shown. Scale bars, 100 µm. ns, not significant. ***p<0.001. (TIF) [file pbio.1001855.s009.tif]
