# Supplementary material for: Tnfa Signaling Through Tnfr2 Protects Skin Against Oxidative Stress–Induced Inflammation
Source: PLoS Biol. 2014 May 6;12(5):e1001855. doi: 10.1371/journal.pbio.1001855 (PMC4011677; doi:10.1371/journal.pbio.1001855)
Supplement: Table S1 — MOs used in this study. The gene symbols followed the Zebrafish Nomenclature Guidelines (http://zfin.org/zf_info/nomen.html). ENA, European Nucleotide Archive (http://www.ebi.ac.uk/ena/). (DOCX) [file pbio.1001855.s010.docx]

| **Gene** | **ENA or Ensembl ID** | **Target** | **Sequence (5’**→**3’)** | **Concentration**  **(mM)** | **Reference** |
| --- | --- | --- | --- | --- | --- |
| *tnfa* | ENSDARG00000009511 | e1/i1 | GCAGGATTTTCACCTTATGGAGCGT | 0.5 | López-Muñoz *et al.*, 2011 |
| *tnfr1* | ENSDARG00000018569 | e6/i6 | CTGCATTGTGACTTACTTATCGCAC | 0.65 | Espín *et al*., 2013 |
| *tnfr2* | ENSDARG00000070165 | i1/e2 | GGAATCTGTGAACACAAAGGGACAA | 0.2 | Espín *et al*., 2013 |
| *duox1* | ENSDARG00000062632 | e8/i8 | AGTGAATTAGAGAAATGCACCTTTT | 0.125 | Niethammer *et al.*, 2009 |
| *p53* | NM_131327 | atg/5'UTR | GCGCCATTGCTTTGCAAGAATTG | 0.1 | Niethammer *et al.*, 2009 |
| *lyn* | ENSDARG00000031715 | e6/i6 | TCAGACAGCAAATAGTAATCACCTT | 0.5 | Yoo *et al.,* 2011 |
| *il1b* | ENSDARG00000005419 | e1/i1 | CCCACAAACTGCAAAATATCAGCTT | 0.6 | López-Muñoz *et al.*, 2011 |
