# Supplementary material for: Tnfa Signaling Through Tnfr2 Protects Skin Against Oxidative Stress–Induced Inflammation
Source: PLoS Biol. 2014 May 6;12(5):e1001855. doi: 10.1371/journal.pbio.1001855 (PMC4011677; doi:10.1371/journal.pbio.1001855)
Supplement: Table S2 — Primers used in this study. The gene symbols followed the Zebrafish Nomenclature Guidelines (http://zfin.org/zf_info/nomen.html). ENA, European Nucleotide Archive (http://www.ebi.ac.uk/ena/). (DOCX) [file pbio.1001855.s011.docx]

| **Gene** | **ENA ID** | **Name** | **Sequence (5’**→**3’)** | **Use** |
| --- | --- | --- | --- | --- |
| *rps11* | NM_213377 | F1 | GGCGTCAACGTGTCAGAGTA | RT-qPCR |
|  |  | R1 | GCCTCTTCTCAAAACGGTTG |  |
| *tnfr1* | NM_213190 | F5 | AGCATTCCCCCAGTCTTTTT |  |
|  |  | R5 | GCAGGTGACGATGACTGAGA |  |
| *tnfr2* | NM_001089510 | F14 | CACACAAGAGATCCGAAGCA |  |
|  |  | R14 | GGCATCTGTGATGGGAACTT |  |
| *tnfa* | NM_212859 | F2 | GCGCTTTTCTGAATCCTACG |  |
|  |  | R2 | TGCCCAGTCTGTCTCCTTCT |  |
| *Il1b* | NM_212844 | F5 | GGCTGTGTGTTTGGGAATCT |  |
|  |  | R5 | TGATAAACCAACCGGGACA |  |
| *duox1* | AB255050 | F | ACACATGTGACTTCATATCCAG |  |
|  |  | R | ATTATTAACTCATCCACATCCAG |  |
| *ptgs2b* | NM_001025504 | F2 | CCCCAGAGTACTGGAAACCA |  |
|  |  | R2 | ACATGGCCCGTTGACATTAT |  |
| *gfp* | EF591490 | F1 | ACGTAAACGGCCACAAGTTC |  |
|  |  | R1 | **AAGTCGTGCTGCTTCATGTG** |  |
| *mpx* | NM_212779 | F1 | AGGGCGTGACCATGCTATAC |  |
|  |  | R1 | AGGCTCAGCAACACCTCCTA |  |
| *krt18* | NM_178437 | F2 | AGAACCTGAAAGGGTCACTGG |  |
|  |  | R2 | GTAGGTGGCGATTTCTGCCT |  |
| *p63* | NM_152986 | F | CTCGGCAAGAACAAACTGCC |  |
|  |  | R | GAGGGGTCACTGAAGGAAGG |  |
